# NanoString-based breast cancer risk prediction for women with sclerosing adenosis

**DOI:** 10.1007/s10549-017-4441-z

**Published:** 2017-08-10

**Authors:** Stacey J. Winham, Christine Mehner, Ethan P. Heinzen, Brendan T. Broderick, Melody Stallings-Mann, Aziza Nassar, Robert A. Vierkant, Tanya L. Hoskin, Ryan D. Frank, Chen Wang, Lori A. Denison, Celine M. Vachon, Marlene H. Frost, Lynn C. Hartmann, E. Aubrey Thompson, Mark E. Sherman, Daniel W. Visscher, Amy C. Degnim, Derek C. Radisky

**Affiliations:** 1 Division of Biomedical Statistics and Informatics, Department of Health Sciences Research, Mayo Clinic, Rochester, MN 55905 USA; 2 Department of Cancer Biology, Mayo Clinic, Jacksonville, FL 32224 USA; 3 Department of Laboratory Medicine and Pathology, Mayo Clinic, Jacksonville, FL 32224 USA; 4 Department of Information Technology, Mayo Clinic, Rochester, MN 55905 USA; 5 Department of Medical Oncology, Mayo Clinic, Rochester, MN 55905 USA; 6 Department of Health Sciences Research, Mayo Clinic, Jacksonville, FL 32224 USA; 7 Department of Laboratory Medicine and Pathology, Mayo Clinic, Rochester, MN 55905 USA; 8 Department of Surgery, Mayo Clinic, Rochester, MN 55905 USA

**Keywords:** Benign breast disease, Sclerosing adenosis, Breast cancer, Formalin-fixed paraffin-embedded, NanoString, Risk prediction

## Abstract

**Purpose:**

Sclerosing adenosis (SA), found in ¼ of benign breast disease (BBD) biopsies, is a histological feature characterized by lobulocentric proliferation of acini and stromal fibrosis and confers a two-fold increase in breast cancer risk compared to women in the general population. We evaluated a NanoString-based gene expression assay to model breast cancer risk using RNA derived from formalin-fixed, paraffin-embedded (FFPE) biopsies with SA.

**Methods:**

The study group consisted of 151 women diagnosed with SA between 1967 and 2001 within the Mayo BBD cohort, of which 37 subsequently developed cancer within 10 years (cases) and 114 did not (controls). RNA was isolated from benign breast biopsies, and NanoString-based methods were used to assess expression levels of 61 genes, including 35 identified by previous array-based profiling experiments and 26 from biological insight. Diagonal linear discriminant analysis of these data was used to predict cancer within 10 years. Predictive performance was assessed with receiver operating characteristic area under the curve (ROC-AUC) values estimated from 5-fold cross-validation.

**Results:**

Gene expression prediction models achieved cross-validated ROC-AUC estimates ranging from 0.66 to 0.70. Performing univariate associations within each of the five folds consistently identified genes *DLK2*, *EXOC6*, *KIT*, *RGS12*, and *SORBS2* as significant; a model with only these five genes showed cross-validated ROC-AUC of 0.75, which compared favorably to risk prediction using established clinical models (Gail/BCRAT: 0.57; BBD-BC: 0.67).

**Conclusions:**

Our results demonstrate that biomarkers of breast cancer risk can be detected in benign breast tissue years prior to cancer development in women with SA. These markers can be assessed using assay methods optimized for RNA derived from FFPE biopsy tissues which are commonly available.

**Electronic supplementary material:**

The online version of this article (doi:10.1007/s10549-017-4441-z) contains supplementary material, which is available to authorized users.

## Introduction

**Fig. 1:**
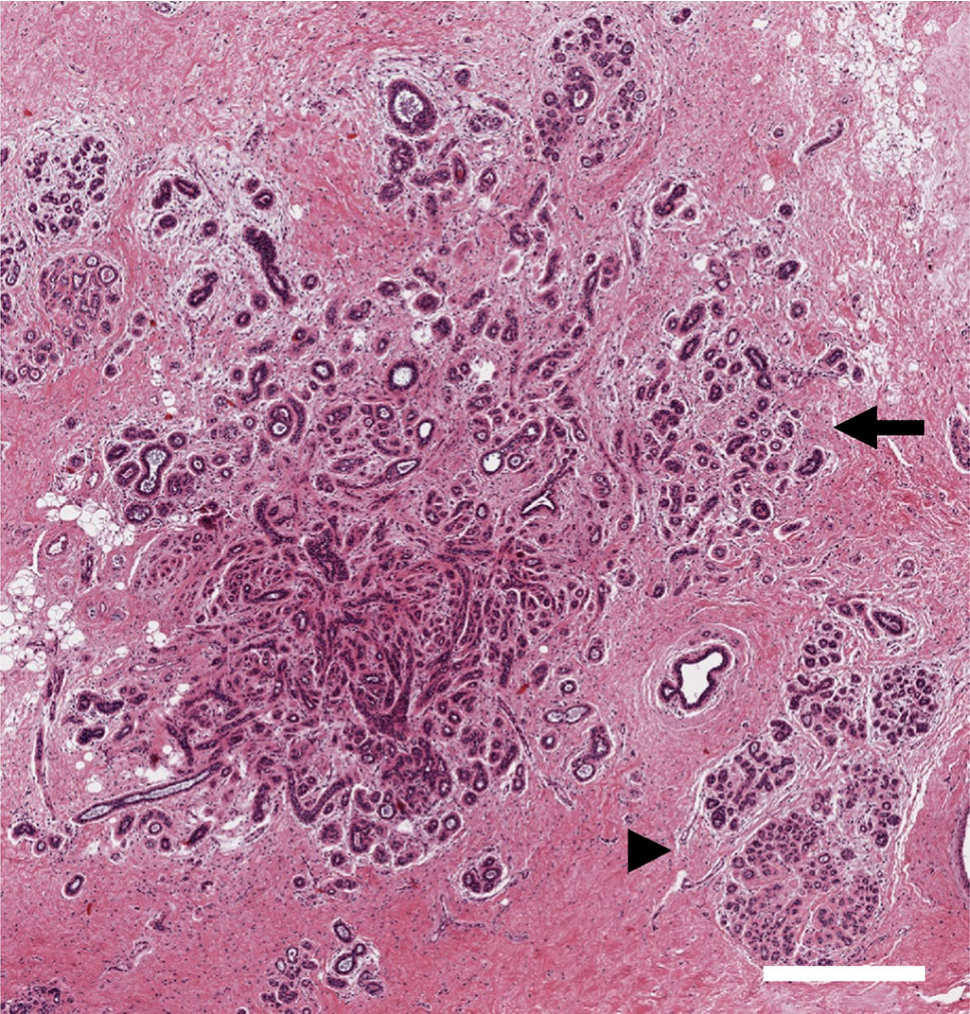
Histology of sclerosing adenosis (SA). H & E image of SA (*arrow*) in field containing normal lobules (*arrowhead*). *Scale bar* 500 μm

Breast cancer (BC) is the most commonly diagnosed cancer in women in the US, with estimated incidence of more than 252,000 new cases and more than 40,000 deaths expected in 2017 [1]. Better identification of which women are at increased risk for developing breast cancer would have considerable benefit for optimal targeting of surveillance and cancer prevention strategies. More than 1 million women in the US have breast biopsies with benign findings every year, and the majority of these biopsies are formalin-fixed and paraffin-embedded (FFPE) to facilitate pathology diagnosis [2, 3]. Investigations within the Mayo Clinic benign breast disease (BBD) cohort have revealed that more than ¼ of the biopsies contain sclerosing adenosis (SA), a histological feature characterized by epithelial and myoepithelial lobulocentric proliferation, disordered acinar architecture, and stromal fibrosis (Fig. 1); women with SA have an approximately doubled risk of subsequent breast cancer development [2, 4, 5]. We previously generated a microarray-based gene signature using RNA obtained from SA-containing biopsies, and found that this signature was associated with subsequent cancer incidence [6]. The results from these experiments suggested that transcriptional elements associated with cancer risk are present many years prior to development of disease, and could be useful in predicting 10-year cancer risk for women with SA. The purpose of the project described here was to develop an expression-based assay method with clinical utility to refine prognostic genes that will allow us to design focused gene expression assays. We used NanoString-based methods optimized for use with FFPE-derived RNA to define a set of transcriptional features that could be used to create a model for assessment of breast cancer risk for women with SA. We also assessed how transcription-based risk assessments compare with and complement existing Gail/BCRAT and BBD-BC risk models for this group of women. The studies presented here provide proof of principle for the use of the NanoString assay as a method for risk prediction for women with SA, and further showed that a reduced subset of the genes was just as effective for identification of high-risk patients. Thus, this study provides a critical step towards improved breast cancer risk prediction specifically for women with SA, and support for future use of this discovery-validation procedure to identify high-risk subgroups of women with other benign breast disease (BBD) pathologies.

## Methods

### Patients and samples

The Mayo BBD Cohort has been previously described [2, 6, 7]. Demographic descriptors and potential breast cancer risk factors were identified via medical record review and from self-response questionnaires. All subjects have given research authorization. All study procedures have been approved by the Mayo Clinic Institutional Review Board. From patients diagnosed by the study pathologist (D.W.V) with sclerosing adenosis (SA) on their biopsy in the Mayo Clinic BBD Cohort with at least 10 years of follow-up time, a case/control set of 151 women was sampled as previously described [6], including 37 patients who subsequently developed cancer within 10 years (cases) and 114 patients that remained cancer free at 10 years (controls). Study sample, demographic, and clinical characteristics are presented in Table 1. Analysis of the case–control set revealed that cases were more likely to have atypical hyperplasia and were older than controls; no other variables were found to be significantly associated with case–control status.

**Table 1:** SA case–control set characteristics

	Unaffected controls (*N* = 114)^a^	Breast cancer cases (*N* = 37)^a^	Total (*N* = 151)^a^	*P* value^b^
Overall impression				0.042
PDWA	101 (88.6%)	27 (73%)	128 (84.8%)	
AH	13 (11.4%)	10 (27%)	23 (15.2%)	
Number of atypical foci				0.107
0	101 (88.6%)	27 (73%)	128 (84.8%)	
1	6 (5.26%)	3 (8.11%)	9 (5.96%)	
2	3 (2.63%)	3 (8.11%)	6 (3.97%)	
3 or more	4 (3.51%)	4 (10.8%)	8 (5.3%)	
Year of index biopsy				0.124
1967–1981	24 (21.1%)	3 (8.11%)	27 (17.9%)	
1982–1991	90 (78.9%)	34 (91.9%)	124 (82.1%)	
Age at index biopsy				<0.001
Mean (SD)	51.1 (10.2)	58.1 (10.8)	52.8 (10.7)	
Q1, Q3	44.2, 58	48, 65	45.5, 61	
Range	20–75	40–78	20–78	
Family history of breast cancer				0.438
None	50 (44.6%)	21 (56.8%)	71 (47.7%)	
Weak	36 (32.1%)	9 (24.3%)	45 (30.2%)	
Strong	26 (23.2%)	7 (18.9%)	33 (22.1%)	
Extent of lobular involution				0.013
None	13 (11.8%)	11 (31.4%)	24 (16.6%)	
Partial	91 (82.7%)	24 (68.6%)	115 (79.3%)	
Complete	6 (5.45%)	0 (0%)	6 (4.14%)	
Columnar alteration				0.644
Absent	11 (9.65%)	2 (5.41%)	13 (8.61%)	
Present	103 (90.4%)	35 (94.6%)	138 (91.4%)	
Radial scars				0.405
Absent	89 (78.1%)	25 (69.4%)	114 (76%)	
Present	25 (21.9%)	11 (30.6%)	36 (24%)	
Age first live birth/No. Children				0.543
<21, 1 or more	26 (24.1%)	9 (25.7%)	35 (24.5%)	
≥21, 3 or more	38 (35.2%)	11 (31.4%)	49 (34.3%)	
≥21, 1–2	32 (29.6%)	8 (22.9%)	40 (28%)	
Nulliparous	12 (11.1%)	7 (20%)	19 (13.3%)	
BMI at biopsy				0.436
≤21	28 (25.9%)	5 (13.5%)	33 (22.8%)	
22–25	38 (35.2%)	14 (37.8%)	52 (35.9%)	
26–29	21 (19.4%)	8 (21.6%)	29 (20%)	
30+	21 (19.4%)	10 (27%)	31 (21.4%)	
Use of HRT				0.590
Never	37 (34.6%)	13 (41.9%)	50 (36.2%)	
Ever	70 (65.4%)	18 (58.1%)	88 (63.8%)	

*PDWA* proliferative disease without atypia, *AH* atypical hyperplasia, *BMI* body mass index, *HRT* hormone replacement therapy

^a^Numbers expressed as *N* (percent) unless otherwise indicated. Due to a small number of missing data for some variables, total may not equal 151

^b^ *Chi* square tests were used for categorical variables and t-tests for continuous variables (age)

### Gene expression analysis

RNA extraction and quality control and DASL experiments were previously described [6]; briefly, RNA was extracted from three sequential five micron sections of FFPE biopsy tissue, the amount and quality of RNA were assessed for QC standards, and extracted RNA was assessed using the Whole Genome DASL assay (Illumina, San Diego, CA). Thirty-five genes in the original sclerosing adenosis time to cancer-10 years (SATTC10) model [6] and twenty-six genes selected for biological relevance (Table 2), along with ten housekeeping genes, were used to create a custom code set for NanoString technology analysis to identify a gene signature subset that could be assessed using the NanoString platform for risk of BC among women with SA. The assay was performed according to manufacturer’s protocol (nCounter XT CodeSet Gene Expression Assay). Briefly, 100 ng of extracted RNA was hybridized with the Reporter CodeSet and Capture ProbeSet for 18 h at 65 °C. Samples were loaded onto the NanoString PrepStation for processing and placed into the nCounter cartridge. The cartridge was transferred to the nCounter digital analyzer for image capture and data acquisition of fluorescent reporters. Measurements were taken at high sensitivity with 555 FOV. Normalization was performed using standard procedures with the NanoString-supplied software. Briefly, sample counts were adjusted by the ratio of their mean, and positive controls were adjusted to the overall mean of positive controls, followed by subtraction of the negative control count. The sample counts were then adjusted depending on the ratio to the overall mean of the housekeeping genes, using linear regression to estimate the adjustment factor for each sample. Correlation between normalized DASL probes and NanoString gene expression values was analyzed by Spearman correlation using the software R.

**Table 2:** Genes in NanoString probeset

	SATTC10 genes
AK5	Adenylate kinase 5
ATP6V0B	ATPase, H+ transporting, lysosomal 21 kDa
CCDC64	Coiled-coil domain containing 64
EXOC6	Exocyst complex component 6
GEMIN2	Gem (nuclear organelle) associated protein 2
GSTA1	Glutathione S-transferase alpha 1
HILPDA	Hypoxia inducible lipid droplet-associated
ITPRIPL1	Inositol 1,4,5-trisphosphate receptor interacting
KCNH3	Potassium voltage-gated channel, subfamily H3
KCTD21	Potassium channel tetramerization domain 21
LARP6	La ribonucleoprotein domain family, member 6
LRRC4B	Leucine rich repeat containing 4B
MAN2B2	Mannosidase, alpha, class 2B, member 2
MIR626	microRNA 626
MTHFD2	Methylenetetrahydrofolate dehydrogenase2
MUC15	Mucin 15, cell surface associated
NAPG	*N*-ethylmaleimide-sensitive factor AP gamma
NDRG3	NDRG family member 3
NPFF	Neuropeptide FF-amide peptide precursor
NPNT	Nephronectin
PELI2	Pellino E3 ubiquitin protein ligase family member 2
PSMB1	Proteasome (prosome, macropain) subunit b1
PTCHD1	Patched domain containing 1
RGS12	Regulator of G-protein signaling 12
RNPS1	RNA binding protein S1, serine-rich domain
RRP15	Ribosomal RNA processing 15 homolog
SLC16A4	Solute carrier family 16, member 4
SORBS2	Sorbin and SH3 domain containing 2
TCEA3	Transcription elongation factor A (SII), 3
TGIF1	TGFB-induced factor homeobox 1
TPCN2	Two pore segment channel 2
TTTY17A	Testis-specific transcript, Y-linked 17A
UFL1	UFM1-specific ligase 1
ZNF540	Zinc finger protein 540
ZNF546	Zinc finger protein 546

	Additional selected genes
BRCA1	BRCA1, DNA repair associated
BTBD11	Ankyrin repeat and BTB/POZ domain-containing
MB21D1	Mab-21 Domain Containing 1 (also C6orf150)
DDR1	Discoidin Domain Receptor Tyrosine Kinase 1
DIAPH3	Diaphanous Related Formin 3
DLK2	Delta Like Non-Canonical Notch Ligand 2
EGR2	Early Growth Response 2
FBXO44	F-Box Protein 44
HMGA1	High Mobility Group AT-Hook 1
HOXB6	Homeobox B6
HSDL1	Hydroxysteroid Dehydrogenase Like 1
ITGA6	Integrin Subunit Alpha 6
KIT	KIT Proto-Oncogene Receptor Tyrosine Kinase
MMP14	Matrix Metallopeptidase 14
MMP17	Matrix Metallopeptidase 17
RAC1	Rho Family, Small GTP Binding Protein Rac1
RBBP4	Retinoblastoma binding protein 4
SENP7	SUMO1/Sentrin Specific Peptidase 7
ST6GALNAC5	ST6 N-Acetylgalactosaminide Alpha-2,6-Sialyltransferase 5
STX2	Syntaxin 2
TNFSF11	Tumor Necrosis Factor Superfamily Member 11 (RANKL)
TNK1	Tyrosine Kinase Non Receptor 1
TRIM2	Tripartite Motif Containing 2
UIMC1	Ubiquitin Interaction Motif Containing 1
USP6NL	USP6 N-Terminal Like
ZRANB3	Zinc Finger RANBP2-Type Containing 3

### Statistical analysis

Quality control procedures and normalization were performed on the NanoString gene expression data using the NanoString nSolver Analysis Software. Probes were re-annotated using the Basic Local Alignment Search Tool (BLAST) to obtain the most current gene annotations. Data were normalized by comparing to positive and negative spike-in controls and to the housekeeping genes, and then transforming the expression values using a log2 transformation. Observed expression values less than the spike-in controls were set to missing. Probes that did not map to the intended gene targets (*N* = 2) were excluded: CCDC64 and ZNF546, or failed in more than 20% of samples (*N* = 13): BRCA1, C6orf150, DIAPH3, GSTA1, HOXB6, HSDL1, KCNH3, MUC15, PTCHD1, ST6GALNAC5, TNFSF11, TTTY17A, ZRANB. Therefore, 46 genes were available for analysis (Supplemental Table 2). Samples that failed in more than 50% of probes were also excluded (*N* = 5). As a technical evaluation of the assay, Spearman correlations were assessed between each DASL probe and corresponding Nanostring probe. Additionally, univariate associations of each NanoString probe to risk of breast cancer within 10 years were evaluated with Wilcoxon rank sum tests. Odds ratios were estimated with logistic regression, both unadjusted and adjusted for age.

The previous analysis utilized a split-sample approach with independent training and validation sets [6]. To improve power for prediction modeling, the full sample was utilized via fivefold cross-validation, where the full sample is randomly split into five equally sized pieces (‘folds’); four-fifths of the sample was used for training and the remaining one fifth was left out for an independent test set, with the process being repeated five times across the five folds. Samples were randomly selected for each of the five folds stratified on case–control status, to require equal distributions of cases and controls across fold. Training and test set sample sizes, and age distributions were summarized across each fold to ensure equal distributions. In each training sample, a diagonal linear discriminant analysis (DLDA) model was built to predict case–control status based on multivariate gene expression, and applied to the samples in the testing fold. Performance in the testing fold was evaluated using ROC-AUC, and average AUC estimates across the five testing folds are reported. Models were constructed using gene expression alone, clinical variables alone (Gail/BCRAT and BBD-BC model predictions), and gene expression and clinical variables together. We developed models using all 65 genes from the entire NanoString panel and using the 35 gene from the previously identified SATTC10 dataset [6] to allow for a comparison between the DASL-derived modeling and the current NanoString-derived predictions. Because we found that not all of the assessed gene expression data from the NanoString assessment correlated with the prior DASL data, we aimed to use a more refined set of genes for prediction using univariate filtering from the set of 61 genes (where genes with Wilcoxon rank sum p values less than or equal to 0.05 in the training sample were retained and evaluated in the testing fold). Additionally, sensitivity analyses were conducted to assess confounding by presence of atypical hyperplasia (AH), by removing samples with AH.

Statistical analysis was carried out using R statistical software version 3.3.1 (https://www.r-project.org).

## Results

We generated a NanoString codeset containing the original 35 genes from the SATTC10 model, along with 26 additional genes selected on the basis of biological relevance to breast cancer development and univariate association with case–control status in the original training set of patients [6] (Table 2), and 10 genes for background normalization. After quality control and re-annotation, 46 genes were available for analysis. Overall, NanoString gene expression was significantly correlated with the expression of at least one corresponding DASL probe for 28 of the 46 genes evaluated (*P* < 0.0006, Supplemental Table 1), displaying moderate reproducibility of the gene expression results between the DASL and NanoString methodologies. Of the 46 genes evaluated, expression of 11 were univariately associated with breast cancer risk at 10 years (*P* < 0.05); 7 were from the SATTC10 gene set, and 4 were biologically relevant candidate genes (Supplemental Table 2).

We used the development of breast cancer at 10 years as the primary end point for model development, using DLDA modeling and five-fold cross-validation. When predictive genes were selected on the basis of univariate association with case status for each fold, the number of probes varied from 6 to 17, with receiver operating characteristic area under the curve (ROC-AUC) values averaging 0.78 over the five training sets, and 0.67 over the five holdout validation sets (Table 3). When the BCRAT/Gail model was applied to these same sets, ROC-AUC values averaged at 0.57 in the training sets and 0.55 in the validation sets; combination of the univariate gene models with the BCRAT/Gail assessments provided significant improvement in training and validation sets to 0.78 and 0.68, respectively. Similar assessment of the BBD-BC model yielded average ROC-AUC values of 0.66 in both training and validation sets, which were improved when combined with the univariate gene models to 0.79 and 0.70 in the training and validation sets, respectively. Modeling approaches that used all 35 genes in the SATTC10 gene set and all the genes in the NanoString codeset produced similar results (Supplemental Tables 3, 4, respectively). Furthermore, sensitivity analyses removing subjects with AH also yielded similar result patterns, although AUC estimates were slightly attenuated (Supplemental Table 5).

**Table 3:** ROC AUC values from the fivefold cross-validation DLDA models

Model	# Probes	Cases:controls	Training				
			Gene expression only	Gail model only	Gene expression and Gail model	BBD-BC model only	Gene expression and BBD-BC model
1	6	29:92	0.81	0.54	0.81	0.61	0.81
2	10	29:91	0.78	0.58	0.78	0.69	0.79
3	10	30:91	0.77	0.61	0.78	0.67	0.78
4	10	30:91	0.76	0.61	0.77	0.69	0.79
5	17	30:91	0.75	0.53	0.75	0.63	0.76
		Average	0.78	0.57	0.78	0.66	0.79

Model	# Probes	Cases:controls	Validation				
			Gene expression only	Gail model only	Gene expression and Gail model	BBD-BC model only	Gene expression and BBD-BC model
1	6	8:22	0.73	0.69	0.76	0.82	0.82
2	10	8:23	0.73	0.54	0.72	0.6	0.74
3	10	7:23	0.64	0.41	0.65	0.61	0.67
4	10	7:23	0.68	0.40	0.66	0.52	0.67
5	17	7:23	0.58	0.72	0.59	0.76	0.58
		Average	0.67	0.55	0.68	0.66	0.70

Gene expression only, model contains only the selected probes; Gail model only, model includes only the BCRAT (Gail) Model predicted risk, Gene expression and Gail model, model includes selected probes plus the BCRAT model predicted risk; BBD-BC model only, model includes only the BBD-BC model predicted risk; Gene expression and BBD-BC model, models includes selected probes plus the BBD-BC model predicted risk

**Fig. 2:**
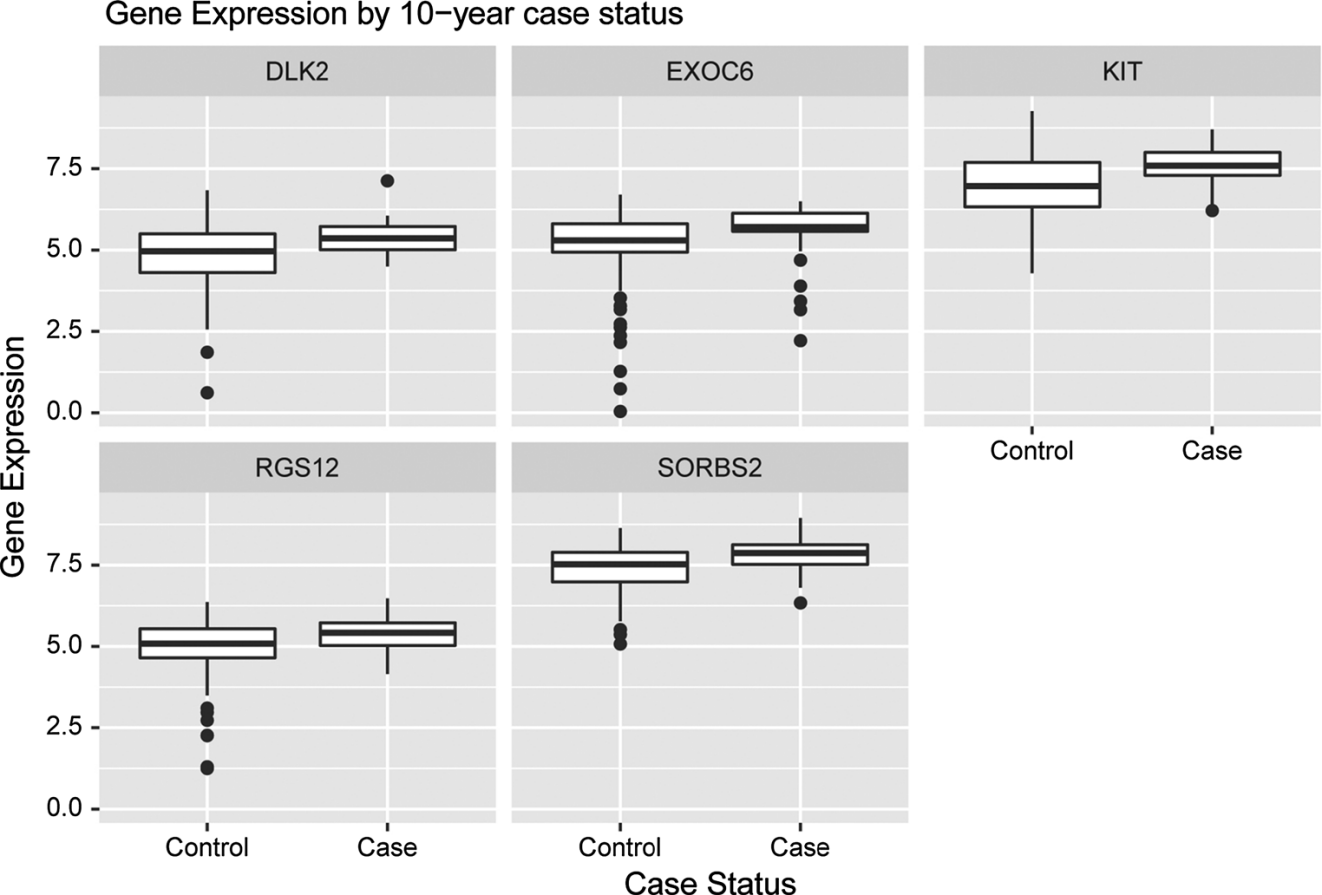
Gene expression distributions of five genes common to all models for breast cancer cases and controls at 10 years post biopsy

When we examined the specific genes selected on the basis of univariate association with case status for each fold, we noted that while there was some variation in gene composition, five genes were present in every fold (Table 4): EXOC6, RGS12, SORBS2 (from the SATTC10 gene set), and DLK2 and KIT (from the set of biologically relevant candidate genes). All of these genes showed higher expression in cases than in controls (Fig. 2), consistent with the positive coefficients for these genes in all models; additionally, a cross-validated model using just these five genes produced a cross-validated ROC-AUC of 0.75, similar to models with more genes (Supplemental Table 6).

**Table 4:** Model training set DLDA coefficients by fold

Higher coefficients indicate higher expression in cases compared to controls; a coefficient of zero indicates no association/absence from the model

We also observed that the ROC-AUCs for the gene prediction models in the leave out validation sets decreased with increasing gene numbers from 0.73 for 6 genes to 0.58 for 17 genes (Table 3). The DLDA method generates regression coefficients for each feature and a corresponding intercept for each model; the magnitude and sign of the coefficients in each model revealed that the relative contribution for each of the five common genes decreased as the models increased in gene number (Table 4), supporting the concept that these genes are most important for prediction, and that additional features decrease classification accuracy.

## Discussion

We show that NanoString-based gene expression data can be used to model cancer risk for women with sclerosing adenosis, a common proliferative finding present in about ¼ of all benign biopsies. The expression analysis used highly fragmented RNA derived from archival FFPE benign tissue biopsies, and yet performed well with the NanoString assay. Models utilizing gene expression data performed better on average than either the BCRAT/Gail or BBD-BC models, and models including both gene expression and clinical predictor variables showed slightly improved performance compared to either gene expression or clinical predictor variables alone. Univariate modeling of randomly selected sets of the genes showed consistent association of five genes with case status, and of these five genes, two are therapeutically targetable cell surface receptors that have been implicated previously in cancer development or progression: DLK2, an effector of the NOTCH signaling pathway [8, 9], and KIT, a receptor for stem cell factor and other signaling molecules that is inhibited by imatinib [10, 11]. That, all models using DLK2 and KIT had positive coefficients for their expression values (indicating increased expression of the markers is associated with increased BC risk, Table 4) is consistent with their generally accepted roles in cancer development and progression. The other three genes that showed consistent expression across all models, SORBS2, RGS12, and EXOC6, have been investigated as predictive or prognostic cancer biomarkers [12–15]. While it is unclear specifically how these latter three genes may contribute to breast cancer development or whether these molecules can be targeted therapeutically, consistently positive coefficients are suggestive of protumorigenic roles. Further investigations will be necessary to evaluate whether these risk markers are specific for women with SA or whether the biomarkers identified here are indicative of differential risk for all women with BBD.

Although the Breast Cancer Risk Assessment Tool (BCRAT, also referred to as the Gail model) [16] provides risk estimates at the population level, it is not as reliable when predicting risk for individual women [17, 18]. An individualized BC risk assessment model, designated the BBD-BC model, was recently developed for women with BBD and includes histologic features of the biopsy, including SA, as well as other demographic and clinical features. The BBD-BC model was found to provide improved performance for women with BBD as compared to the BCRAT model [18]. For women with SA in particular, we have found that risk stratification can be achieved by consideration of other histological and clinical features as well as expression of the proliferation marker Ki-67 [5, 19]. Our results here show that further improvements in individualized risk prediction can be obtained through examination of transcriptional biomarkers expressed in the benign breast biopsy tissue [19–25].

Microarray-based gene expression platforms have been instrumental for advancing our understanding of breast cancer and treatment and for identifying prognostic and predictive gene signatures [26]. Although microarray-based methods work well with RNA derived from fresh or frozen samples, their mostly poorer performance with the highly fragmented RNA that is derived from FFPE biopsies has delayed their broad clinical implementation [27]. NanoString nCounter analysis methods quantify immobilized RNA using customized barcodes; because this method does not require library generation or polymerase action, it works well with FFPE-derived RNA. Compared to assays requiring fresh/frozen tissue, FFPE-based assays facilitate clinical implementation since no changes in sample collection and processing are needed. Our results presented here validate the feasibility of our overall goal to create an assay that incorporates NanoString-derived gene expression biomarkers with patient demographic information and pathological characteristics of the benign biopsy that can be applied to all women diagnosed with SA. Significant improvements in prediction ability will require application of these methods to larger patient cohorts and validation across multiple patient populations.

Strengths of our study include our focus on a SA, which is diagnosed in as many as 250,000–500,000 women per year in the United States; since SA is associated with a more than doubling of BC risk, the aggregate increased BC incidence following SA diagnosis is substantial. Moreover, unlike very high-risk lesions such as atypical hyperplasia, there are currently no clinical recommendations for women diagnosed with SA and no way to assess which of these women are at high risk and could thus benefit from interventions to reduce future BC incidence. Additionally, because SA is a cellular and homogenous lesion that can represent a substantial area of the biopsy tissue section, it represents an optimal target for methods that can identify risk signatures from RNA derived from entire tissue sections of FFPE samples, an approach that we feel will be necessary for broadest clinical translation for this patient population. Use of a NanoString-based risk signature assay offers the advantage of objective risk data and is independent of pathology interpretation. Moreover, our assay provides additional risk stratification when combined with standard clinical models, although as noted above, additional studies using larger patient cohorts will be necessary to optimally combine clinical information with transcriptional biomarkers for assessment of BC risk in patients with SA. The threshold used to determine case status from the predicted score can be optimized in future studies to reduce false positives and false negatives while balancing the consequences of each. Our study uses RNA derived from whole tissue sections rather than laser microdissected lesions; we believe this is most appropriate, since subsequent cancers derive from the complex tissue microenvironment, in which stromal factors are increasingly recognized as important in cancer progression [28]. Limitations include our focus on SA, which limits the application of our signature to this particular patient group, the relatively small number of events in each of the fivefold cross validations, the absence of a completely independent validation set and limited generalizability to women of European descent. Furthermore, our cases and controls were not matched, resulting in cases that were older and more likely to have atypia hyperplasia than controls; although this could induce potential biases, results were not substantially different when adjusted for clinical model predictions (which include age) or when restricted to subjects without atypia. Additionally, our approach used linear modeling to clarify application and interpretation; more sophisticated modeling methods that incorporate higher level feature interaction might further improve risk prediction, but these would require larger patient sample sizes.

In conclusion, we have found that the relative expression levels of a small set of genes, determined from RNA derived from FFPE-banked tissue biopsies and quantified using a clinically relevant transcriptional assay method, can be used to assess breast cancer risk for women with SA, which is found in more than 250,000 women per year in the US alone. Our results also identify specific genes that may influence breast cancer development, and thus represent potential targets for novel intervention strategies. Ultimate clinical translation of our approach will aid in decision-making for women with SA and their physicians, who would be better able to choose prevention strategies for women predicted to be at higher risk, and watchful waiting for those women predicted to be in lower risk categories [10, 11].

## Electronic supplementary material

Below is the link to the electronic supplementary material.

Supplementary material 1 (PDF 134 kb)
