# Supplementary material for: NanoString-based breast cancer risk prediction for women with sclerosing adenosis
Source: Breast Cancer Res Treat. 2017 Aug 10;166(2):641–50. doi: 10.1007/s10549-017-4441-z (PMC5668350; doi:10.1007/s10549-017-4441-z)
Supplement: Supplementary file 1 — Supplementary material 1 (PDF 134 kb) [file 10549_2017_4441_MOESM1_ESM.pdf]

Supplemental Table 1. DASL NanoString correlations

DASL probe ID	Nanostrong Gene ID	Correlation[1]	P-value	P<0.05	P<0.0006
ILMN_1656593	AK5	0.480	8.00E-10	1	1
ILMN_2283473	AK5	0.280	5.40E-04	1	1
ILMN_2387712	AK5	0.660	2.70E-19	1	1
ILMN_1721391	ATP6V0B	0.330	5.20E-05	1	1
ILMN_2353642	ATP6V0B	0.180	2.90E-02	1	0
ILMN_1674670	BTBD11	-0.300	2.30E-04	1	1
ILMN_1696582	BTBD11	-0.056	5.00E-01	0	0
ILMN_1705066	BTBD11	0.130	1.10E-01	0	0
ILMN_1812262	DDR1	0.099	2.30E-01	0	0
ILMN_2360054	DDR1	-0.240	4.00E-03	1	0
ILMN_2290547	DDR1	0.031	7.10E-01	0	0
ILMN_1662010	DLK2	-0.180	2.70E-02	1	0
ILMN_1676728	DLK2	0.180	3.10E-02	1	0
ILMN_1702943	DLK2	0.088	2.90E-01	0	0
ILMN_1743199	EGR2	0.056	5.00E-01	0	0
ILMN_1667686	EXOC6	-0.400	8.80E-07	1	1
ILMN_1651628	EXOC6	-0.300	2.90E-04	1	1
ILMN_1679687	EXOC6	0.076	3.60E-01	0	0
ILMN_1732182	FBXO44	0.210	9.60E-03	1	0
ILMN_2325625	FBXO44	0.120	1.40E-01	0	0
ILMN_1651597	FBXO44	0.100	2.20E-01	0	0
ILMN_2344002	GEMIN2	0.300	2.80E-04	1	1
ILMN_2344007	GEMIN2	0.190	2.00E-02	1	0
ILMN_1659990	HILPDA	-0.130	1.10E-01	0	0
ILMN_1693560	HMGA1	0.430	8.30E-08	1	1
ILMN_1742699	HMGA1	0.420	2.60E-07	1	1
ILMN_2311537	HMGA1	0.070	4.10E-01	0	0
ILMN_2359287	ITGA6	-0.180	2.90E-02	1	0
ILMN_2267488	ITGA6	0.450	1.60E-08	1	1
ILMN_1798373	ITPRIPL1	0.170	3.80E-02	1	0
ILMN_1788240	ITPRIPL1	0.048	5.70E-01	0	0
ILMN_1772383	ITPRIPL1	-0.089	2.90E-01	0	0
ILMN_1809708	KCTD21	0.090	2.80E-01	0	0
ILMN_1790160	KIT	0.640	0.00E+00	1	1
ILMN_2229379	KIT	0.430	1.00E-07	1	1
ILMN_1752810	LARP6	0.180	2.60E-02	1	0
ILMN_1663401	LARP6	0.360	7.00E-06	1	1
ILMN_1770479	LMO7	0.290	4.50E-04	1	1
ILMN_2297196	LRRC4B	0.300	2.00E-04	1	1
ILMN_1768510	MAN2B2	0.170	4.70E-02	1	0
ILMN_1774739	MMP14	-0.290	5.30E-04	1	1
ILMN_1801146	MMP17	0.064	4.50E-01	0	0
ILMN_1674706	MTHFD2	0.420	1.80E-07	1	1
ILMN_2293322	MTHFD2	-0.200	1.70E-02	1	0
ILMN_2405521	MTHFD2	0.410	5.50E-07	1	1
ILMN_1788268	NAPG	-0.290	3.80E-04	1	1
ILMN_1738229	NDRG3	-0.170	3.90E-02	1	0
ILMN_2385097	NDRG3	-0.300	3.00E-04	1	1

ILMN_1682503	NPFF	-0.062	4.60E-01	0	0
ILMN_1735762	NPNT	-0.028	7.40E-01	0	0
ILMN_1780132	PELI2	0.380	2.70E-06	1	1
ILMN_1789176	PSMB1	-0.310	1.60E-04	1	1
ILMN_2359789	RAC1	-0.072	3.90E-01	0	0
ILMN_1652445	RAC1	-0.200	1.50E-02	1	0
ILMN_3251085	RBBP4	0.420	2.30E-07	1	1
ILMN_2062524	RBBP4	0.340	3.20E-05	1	1
ILMN_1751141	RGS12	0.230	4.90E-03	1	0
ILMN_1722834	RGS12	0.640	3.70E-18	1	1
ILMN_2384629	RGS12	-0.110	1.80E-01	0	0
ILMN_1691843	RNPS1	-0.410	3.90E-07	1	1
ILMN_2375386	RNPS1	-0.310	1.30E-04	1	1
ILMN_1685661	RRP15	0.260	1.60E-03	1	0
ILMN_1778294	SENP7	0.390	1.70E-06	1	1
ILMN_2382354	SENP7	0.420	2.20E-07	1	1
ILMN_2102069	SLC16A4	0.290	3.80E-04	1	1
ILMN_1804673	SLC16A4	0.460	7.50E-09	1	1
ILMN_1693183	SORBS2	0.089	2.90E-01	0	0
ILMN_1716407	SORBS2	0.530	0.00E+00	1	1
ILMN_2407879	SORBS2	0.480	1.20E-09	1	1
ILMN_1674098	SORBS2	-0.130	1.20E-01	0	0
ILMN_1726805	STX2	-0.200	1.50E-02	1	0
ILMN_1747775	STX2	0.170	4.00E-02	1	0
ILMN_2344216	STX2	0.450	2.90E-08	1	1
ILMN_1726928	TCEA3	0.570	9.50E-14	1	1
ILMN_1754102	TGIF1	0.130	1.30E-01	0	0
ILMN_2247842	TGIF1	-0.013	8.80E-01	0	0
ILMN_2318643	TGIF1	0.310	1.40E-04	1	1
ILMN_1702211	TGIF1	0.043	6.10E-01	0	0
ILMN_2318638	TGIF1	0.680	0.00E+00	1	1
ILMN_2245292	TGIF1	-0.210	1.00E-02	1	0
ILMN_1656761	TGIF1	-0.470	3.40E-09	1	1
ILMN_1724096	TNK1	0.320	1.10E-04	1	1
ILMN_1726873	TPCN2	0.065	4.40E-01	0	0
ILMN_1745079	TRIM2	0.079	3.50E-01	0	0
ILMN_2058975	UFL1	-0.110	1.80E-01	0	0
ILMN_1691188	UIMC1	0.009	9.20E-01	0	0
ILMN_2311826	USP6NL	0.075	3.70E-01	0	0
ILMN_1659696	USP6NL	0.150	7.00E-02	1	0
ILMN_1681846	ZNF540	0.400	6.50E-07	1	1

[1]Spearman's rank correlation coefficients (rho).
[2]Indicator if p-value is less than the uncorrected threshold.
[3]Indicator if p-value is less than the Bonferroni corrected threshold for 46 probes.

Supplemental Table 2. Univariate associations of gene expression values by breast cancer status at 10 years.

Gene	Mean Expression Cases	Mean Expression Controls	Wilcoxon P-value	OR	Age-adjusted OR	In SATTC10?
AK5	7.843	7.252	0.008	1.714	1.426	Yes
ATP6V0B	8.534	8.581	0.578	0.867	0.707	Yes
BTBD11	4.795	4.453	0.119	1.674	1.749	No
DDR1	7.840	7.613	0.013	2.941	2.220	No
DLK2	5.367	4.831	0.002	2.331	2.136	No
EGR2	6.393	6.310	0.895	1.074	1.211	No
EXOC6	5.554	5.121	0.001	1.675	1.565	Yes
FBXO44	7.715	7.585	0.235	1.653	1.265	No
GEMIN2	4.956	4.821	0.504	1.125	1.064	Yes
HILPDA	6.735	6.635	0.427	1.158	1.037	Yes
HMGA1	8.111	8.111	0.526	1.000	0.992	No
ITGA6	7.969	7.755	0.015	3.616	3.854	No
ITPRIPL1	4.357	3.962	0.091	1.584	1.449	Yes
KCTD21	5.854	5.567	0.098	1.684	1.269	Yes
KIT	7.564	6.968	0.001	2.261	2.278	No
LARP6	5.598	5.204	0.188	1.503	1.426	Yes
LMO7	7.084	6.984	0.648	1.266	1.265	No
LRRC4B	3.905	4.460	0.006	0.621	0.618	Yes
MAN2B2	7.520	7.447	0.546	1.334	0.975	Yes
MMP14	7.568	7.258	0.201	1.360	1.515	No
MMP17	5.074	5.061	0.314	1.019	0.918	No
MTHFD2	6.858	6.629	0.358	1.335	1.256	Yes
NAPG	6.064	5.799	0.117	1.474	1.354	Yes
NDRG3	7.171	7.086	0.817	1.296	1.283	Yes
NPFF	5.379	5.518	0.168	0.721	0.706	Yes
NPNT	6.643	6.221	0.103	1.402	1.208	Yes
PELI2	5.455	5.264	0.626	1.207	1.327	Yes
PSMB1	6.881	6.458	0.026	1.797	1.528	Yes
RAC1	9.108	9.043	0.810	1.250	0.966	No
RBBP4	7.132	6.867	0.137	1.744	1.635	No
RGS12	5.376	4.966	0.011	2.273	2.085	Yes
RNPS1	8.644	8.497	0.073	2.314	1.717	Yes
RRP15	5.429	5.144	0.189	1.748	1.807	Yes
SENP7	7.685	7.474	0.271	1.486	1.433	No
SLC16A4	5.156	4.847	0.173	1.549	1.359	Yes
SORBS2	7.816	7.391	0.002	3.130	2.409	Yes
STX2	6.129	6.161	0.310	0.964	0.875	No
TCEA3	7.221	6.868	0.106	1.610	1.321	Yes
TGIF1	7.863	7.629	0.049	1.979	1.726	Yes
TNK1	6.210	5.947	0.060	1.777	1.395	No
TPCN2	7.167	7.028	0.437	1.410	1.290	Yes
TRIM2	5.756	5.897	0.273	0.695	0.605	No
UFL1	8.627	8.640	0.741	0.871	0.846	Yes
UIMC1	6.888	6.955	0.163	0.822	0.683	No
USP6NL	7.483	7.434	0.981	1.240	1.134	No
ZNF540	5.598	5.368	0.443	1.312	1.138	Yes

Mean Expression Cases, mean of gene expression across case samples; Mean Expression Controls, mean of gene expression across control samples; Wilcoxon P-value, p-value from a Wilcoxon Rank Sum Test comparing mean gene expression in cases vs. controls; OR, odds ratio from a logistic regression model predicting case-control status; age-adjusted OR, odds ratio from a logistic regression model adjusting for age; In SA-TTC10, indicator of whether the probe was part of the previously derived SA-TTC10 model or selected based on biological insight.

Supplemental Table 3. ROC AUC values from the 5-fold cross-validation using the genes in the SATTC10 gene set

		Training					
**fold**	**# probes**	**cases:controls**	**Gene expression only**	**Gail model only**	**Gene expression and Gail model**	**BBD-BC model only**	**Gene expression and BBD-BC model**
1	27	29:92	0.67	0.57	0.68	0.67	0.70
2	27	29:91	0.65	0.52	0.65	0.62	0.66
3	27	30:91	0.67	0.60	0.68	0.69	0.70
4	27	30:91	0.71	0.57	0.71	0.63	0.72
5	27	30:91	0.72	0.59	0.72	0.67	0.73
		**average**	**0.69**	**0.57**	**0.69**	**0.65**	**0.70**

		Validation					
**fold**	**# probes**	**cases:controls**	**Gene expression only**	**Gail model only**	**Gene expression and Gail model**	**BBD-BC model only**	**Gene expression and BBD-BC model**
1	27	8:22	0.74	0.57	0.74	0.60	0.76
2	27	8:23	0.86	0.82	0.86	0.81	0.88
3	27	7:23	0.79	0.42	0.77	0.54	0.76
4	27	7:23	0.53	0.60	0.54	0.76	0.56
5	27	7:23	0.39	0.45	0.39	0.57	0.40
		**average**	**0.66**	**0.57**	**0.66**	**0.66**	**0.67**

Genes expression only, model contains only the selected probes; Gail model only, model includes only the the BCRAT (Gail) Model predicted risk, Gene expression and Gail model, model includes selected probes plus the BCRAT model predicted risk; BBD-BC model only, model includes only the BBD-BC model predicted risk; Gene expression and BBD-BC model, models includes selected probes plus the BBD-BC model predicted risk.

Supplemental Table 4. ROC AUC values from the 5-fold cross-validation using all genes in the Nanostring codeset

		Training					
fold	# probes	cases: controls	Gene expression only	Gail model only	Gene expression and Gail model	BBD-BC model only	Gene expression and BBD-BC model
1	46	29:92	0.76	0.56	0.76	0.67	0.76
2	46	29:91	0.68	0.59	0.69	0.69	0.70
3	46	30:91	0.73	0.61	0.73	0.66	0.73
4	46	30:91	0.73	0.52	0.73	0.60	0.73
5	46	30:91	0.72	0.59	0.71	0.67	0.72
		**average**	**0.72**	**0.57**	**0.72**	**0.66**	**0.73**

		Validation					
fold	# probes	cases: controls	Gene expression only	Gail model only	Gene expression and Gail model	BBD-BC model only	Gene expression and BBD-BC model
1	46	8:22	0.59	0.63	0.59	0.64	0.60
2	46	8:23	0.92	0.54	0.89	0.53	0.90
3	46	7:23	0.53	0.42	0.55	0.69	0.57
4	46	7:23	0.69	0.76	0.69	0.86	0.70
5	46	7:23	0.77	0.49	0.77	0.61	0.77
		**average**	**0.70**	**0.57**	**0.70**	**0.66**	**0.71**

Gene expression only, model contains only the selected probes; Gail model only, model includes only the BCRAT (Gail) Model predicted risk, Gene expression and Gail model, model includes selected probes plus the BCRAT model predicted risk; BBD-BC model only, model includes only the BBD-BC model predicted risk; Gene expression and BBD-BC model, models includes selected probes plus the BBD-BC model predicted risk.

Supplemental Table 5. Sensitivity analysis models without atypia samples.

		Training					
**fold**	**# probes**	**cases:controls**	**Gene expression only**	**Gail model only**	**Gene expression and Gail model**	**BBD-BC model only**	**Gene expression and BBD-BC model**
1	46	26:81	0.71	0.53	0.71	0.63	0.72
2	46	26:81	0.78	0.53	0.78	0.60	0.78
3	46	26:81	0.75	0.55	0.75	0.62	0.75
4	46	26:81	0.72	0.54	0.72	0.60	0.73
5	46	26:81	0.75	0.49	0.75	0.61	0.75
		**average**	**0.74**	**0.53**	**0.74**	**0.61**	**0.74**

		Validation					
**fold**	**# probes**	**cases:controls**	**Gene expression only**	**Gail model only**	**Gene expression and Gail model**	**BBD-BC model only**	**Gene expression and BBD-BC model**
1	46	6:20	0.82	0.51	0.82	0.52	0.82
2	46	6:20	0.66	0.55	0.66	0.68	0.66
3	46	5:20	0.62	0.49	0.58	0.60	0.58
4	46	5:20	0.55	0.46	0.55	0.71	0.54
5	46	5:21	0.71	0.64	0.72	0.59	0.69
		**average**	**0.67**	**0.53**	**0.67**	**0.62**	**0.66**

Genes expression only, model contains only the selected probes; Gail model only, model includes only the BCRAT (Gail) Model predicted risk, Gene expression and Gail model, model includes selected probes plus the BCRAT model predicted risk; BBD-BC model only, model includes only the BBD-BC model predicted risk; Gene expression and BBD-BC model, models includes selected probes plus the BBD-BC model predicted risk.

Supplemental Table 6. ROC AUC values from the 5-fold cross-validation using the five genes present in all models

		Training					
fold	# probes	cases:controls	Gene expression only	Gail model only	Gene expression and Gail model	BBD-BC model only	Gene expression and BBD-BC model
1	5	29:91	0.78	0.61	0.79	0.70	0.82
2	5	29:91	0.76	0.54	0.76	0.65	0.79
3	5	30:90	0.77	0.58	0.78	0.67	0.80
4	5	30:90	0.77	0.60	0.78	0.67	0.79
5	5	30:90	0.74	0.53	0.74	0.59	0.76
		**average**	**0.77**	**0.57**	**0.77**	**0.65**	**0.79**

		Validation					
fold	# probes	cases:controls	Gene expression only	Gail model only	Gene expression and Gail model	BBD-BC model only	Gene expression and BBD-BC model
1	5	8:22	0.69	0.39	0.66	0.53	0.66
2	5	8:22	0.73	0.66	0.75	0.67	0.77
3	5	7:23	0.73	0.58	0.75	0.60	0.75
4	5	7:23	0.75	0.47	0.75	0.61	0.80
5	5	7:23	0.88	0.75	0.88	0.91	0.93
		**average**	**0.75**	**0.57**	**0.76**	**0.67**	**0.78**

Genes expression only, model contains only the selected probes; Gail model only, model includes only the BCRAT (Gail) Model predicted risk, Gene expression and Gail model, model includes selected probes plus the BCRAT model predicted risk; BBD-BC model only, model includes only the BBD-BC model predicted risk; Gene expression and BBD-BC model, models includes selected probes plus the BBD-BC model predicted risk.
